# Ocular Surface Health in Myopic School‐Age Children: An Observational Study

**DOI:** 10.1155/joph/9197399

**Published:** 2025-12-15

**Authors:** Haiyun Wang, Yanwen Zheng, Guang Wang, Limin Bu, Jiwen Yang

**Affiliations:** ^1^ The Optometry Center of Liaoning Aier Eye Hospital, Shenyang, Liaoning, China

**Keywords:** dry eye, myopia, ocular surface, OSDI questionnaire, schoolchildren, tear film

## Abstract

**Objective:**

To examine the clinical characteristics of tear film and ocular surface parameters in schoolchildren with myopia.

**Methods:**

This prospective study included 224 myopic schoolchildren aged 7–14 years. The Ocular Surface Disease Index (OSDI) questionnaire was administered, and tear volume was assessed using a strip meniscometry tube (SMTube) and Schirmer I test. An IDRA ocular surface analyzer (SBM Sistemi, Inc., Torino, Italy) measured the noninvasive first break time (NIBUTf), mean break time (NIBUTav), tear meniscus height (TMH), mean lipid layer thickness (LLTav), meibomian gland atrophy (MGA), blink frequency (BF), and blink quality (BQ). Differences in ocular surface parameters by sex, age, spherical equivalent (SE), and OSDI scores were analyzed.

**Results:**

Median values of ocular surface parameters were SMTube 6.0 mm, Schirmer I 17.0 mm, NIBUTf 7.4 s, NIBUTav 10.1 s, TMH 0.21 mm, LLTav 64 nm, superior MGA (Sup‐MGA) 33%, inferior MGA (Inf‐MGA) 21.5%, BF 4.1s, and BQ 100%. Significant differences in NIBUTf, NIBUTav, and LLTav were observed between the sexes (*p* < 0.05), while TMH differed by the age group (*p* = 0.001). Age, SMTube, and Schirmer values varied significantly with OSDI scores (*p* < 0.05). No significant differences in tear parameters were found among myopia groups. Correlation analysis revealed that SMTube positively correlated with Schirmer (*r* = 0.6236), NIBUTf (*r* = 0.1585), NIBUTav (*r* = 0.1931), and TMH (*r* = 0.0093) (all *p* < 0.005).

**Conclusion:**

Nearly one‐fourth of myopic schoolchildren have dry eye symptoms, primarily due to the reduced tear volume. Age and sex significantly influenced tear film quality and quantity, while myopia severity showed no significant association.

## 1. Background

Ocular surface integrity in school‐aged children is increasingly recognized as a significant public health issue [1]. It is vital not only for visual comfort but also for visual function, academic performance, and overall quality of life. During childhood, the ocular surface system remains under development and is particularly susceptible to disruptions from increased near‐work activities and prolonged digital screen exposure [2]. These factors can impose additional strain, leading to decreased blink rate, compromised tear film stability, and accelerated tear evaporation, ultimately contributing to dry eye and visual fatigue symptoms [2, 3].

Concurrently, the global rise in childhood myopia has spurred investigation into its multifactorial pathogenesis [4, 5]. Emerging evidence indicates a bidirectional relationship between ocular surface dysfunction and myopia progression. For example, an unstable tear film may cause blurred vision and asthenopia, potentially inducing accommodative spasm and stimulating axial elongation. Conversely, standard myopia control interventions—such as orthokeratology, low‐concentration atropine eye drops, or multifocal soft contact lenses—may themselves disrupt the ocular surface microenvironment by altering tear dynamics, corneal epithelial integrity, or meibomian gland function [6]. Although mechanistic hypotheses continue to evolve, robust causal evidence linking these factors remains limited and merits further investigation.

Clinical evaluation of ocular surface health in children poses distinct challenges [7, 8], largely due to the absence of pediatric‐specific diagnostic criteria and the reliance on adult‐based protocols that inadequately account for developmental and behavioral characteristics. Conventional tests such as Schirmer’s test and corneal fluorescein staining are invasive and often poorly tolerated in pediatric populations, reducing their reliability. Moreover, young children may have difficulty articulating symptoms such as “dryness” or “foreign body sensation,” frequently presenting instead with behavioral signs such as eye rubbing, frequent blinking, photophobia, or inattention—further complicating early diagnosis.

Given these challenges, there is a pressing need for systematic studies on ocular surface health in school‐aged children. This study aims to collect both subjective and objective ocular surface metrics in myopic children using the Ocular Surface Disease Index (OSDI) questionnaire, supplemented by functional assessments including strip meniscometry tube (SMTube), Schirmer I test, and the IDRA ocular surface analyzer (SBM Sistemi, Turin, Italy). We intend to characterize clinical ocular surface parameters and evaluate their association with sex, age, and myopia severity. The findings are expected to inform evidence‐based guidelines for assessing ocular surface health in children, facilitate early intervention, and support personalized management strategies for long‐term ocular health.

## 2. Study Object and Methods

### 2.1. Object of Study

This prospective descriptive study adhered to the principles of the Declaration of Helsinki and received approval from the Ethics Committee of Liaoning Aier Eye Hospital (2023‐012‐01). Written informed consent was obtained from the parents or guardians of all participants. The study included myopic schoolchildren aged 7–14 years treated at the Optometry Center of Liaoning Aier Eye Hospital between November 2023 and May 2024. All subjects were examined for detailed medical history, comprehensive eye examinations, OSDI questionnaire, SMTube, Schirmer I test, and IDRA examination of the eye surface interferometer.

Inclusion criteria are as follows: 1. normal findings on slit lamp examination; 2. ability to cooperate with the questionnaire; and 3. no prior eye or systemic medical history. Exclusion criteria are as follows: 1. ocular inflammation, infection, surgery, or trauma within the past 6 months; 2. contact lens use within the past month; 3. ocular or systemic use of drugs affecting ocular surface health within the past week, and 4. inability to cooperate with examinations.

### 2.2. Inspection Items and Processes

All participants underwent systematic ophthalmological examinations, including uncorrected and corrected vision, intraocular pressure, comprehensive optometry, and slit lamp microscopy. Further examinations included the OSDI questionnaire, measurement of tear secretion using the SMTube and Schirmer I test, tear film, meibomian glands, and blink analysis by the IDRA ocular surface analyzer. All examinations were conducted by trained ophthalmologists and technicians. Participants first completed systematic ophthalmological evaluations. Dry eye examinations were performed after a 30‐min rest period, ensuring a minimum interval of 20 min between the IDRA eye surface analysis and the SMTube or Schirmer I tests.

#### 2.2.1. OSDI Questionnaire

The standard OSDI questionnaire was retained without modification. To ensure age‐appropriate comprehension, a trained examiner verbally administered each item using simplified language and concrete examples (e.g., “Does it ever feel like there is sand or dirt in your eyes?” for “gritty sensation”). The child was first invited to describe their subjective experience. The parent then supplemented with behavioral observations. Through this collaborative process, a consensus response was reached for each item, reflecting a unified assessment of the child’s symptoms.

Final score = (sum of scores × 25)/(number of questions answered) [9]. The OSDI scale scores were classified as 0–12 (Grade 0: Asymptomatic), 13–22 (Grade 1: Mild Symptoms), 23–32 (Grade 2: Moderate Symptoms), and 33–100 (Grade 3: Severe Symptoms).

#### 2.2.2. IDRA Eye Surface Analyzer (SBM SISTEMI, Inc., Torino, Italy)

The IDRA ocular surface analyzer, an all‐in‐one device introduced in 2018, was employed to automatically assess a comprehensive set of ocular surface parameters, including those related to the tear film and meibomian glands. It provides data on lipid layer thickness (LLT), noninvasive tear film breakdown time (NIBUT), tear meniscus height (TMH), meibomian gland atrophy (MGA), and blink analysis [10].

The IDRA ocular surface analyzer performs automatic self‐calibration upon startup per the manufacturer’s protocol. Additionally, we conducted daily manual calibration checks using internal reference standards to ensure measurement accuracy and consistency throughout the study. To ensure comfort and reliability in children, we made the following adaptations: The built‐in fixation target was employed to maintain engagement, with stimulus luminance set to the minimum level needed for clear measurement to minimize glare, the “quick acquisition” mode was used in NIBUT and TMH measurement to shorten fixation time and accommodate shorter attention spans, and all tests were performed in a dimly lit room to reduce anxiety and environmental interference.

To minimize potential confounding effects on subsequent measurements, testing followed this chronological order: blink analysis, NIBUT, TMH, LLA, and meibomicrography.

For blink pattern analysis, participants were recorded blinking freely for 20 s. IDRA automatically detects and analyzes blink frequency (BF) and blink quality (BQ), displaying the number of full and partial blinks and the BF in the digital form. BF was calculated as 20 s/total number of blinks, and (BQ) was defined as full blinks/total number of blinks. Partial blinks were defined as blinks without touching the upper and lower eyelids [11].

NIBUT and TMH were measured using the Placido ring technique. NIBUT was assessed without fluorescein dye after the participant blinked three times in succession and then kept the eyes open; this process was repeated three times, and the average was recorded. TMH was measured at the lower edge of the eyelid, just below the pupil.

LLT was measured via interferometry, where the interference pattern and color of the lipid tear film were analyzed. The mean, maximum, and minimum LLT values were recorded using an international rating scale although only the mean value was analyzed in this study.

Infrared meibomian photographs were taken, and MGA was calculated as the percentage of MGA relative to the total meibomian area of the eyelid [12].

#### 2.2.3. SMTube

The SMTube test was administered by trained examiners under standardized conditions. All procedures were carried out using a slit lamp set at its lowest illumination setting. During the measurement, the patient was instructed to gaze horizontally toward the nasal side (ideally with a fixation target provided). The slit lamp beam was positioned at the temporal one‐third of the palpebral fissure, taking care not to direct the beam onto the cornea to avoid stimulation. The tip of the test strip was inserted parallel to the eyelid margin into the tear meniscus at the temporal one‐third region, avoiding contact with the bulbar conjunctiva. Throughout the 5‐s measurement period, blinking and eye movement had to be avoided to prevent dislodgement of the strip from the tear meniscus or contact with the cornea, either of which could have compromised measurement accuracy and caused patient discomfort.

#### 2.2.4. Schirmer I Test

Tear production was evaluated under local anesthesia. We applied one drop of 0.5% proparacaine hydrochloride to the lower fornix of each eye. The drop was administered using a sterile, single‐use minim. A waiting period of 60 s was observed after instillation to ensure adequate corneal and conjunctival anesthesia before the insertion of the Schirmer test tapes.

A standard 5 × 40 mm Schirmer tape was folded at the incision, with the folded end hooked over the temporal third of the lower eyelid margin. Participants were asked to gently close their eyes for 5 min, and the wetting distance from the incision was recorded.

### 2.3. Statistical Method

Statistical analyses were conducted using SPSS (version 24.0). Normality and homogeneity of variances were assessed using the Shapiro–Wilk test and Levene’s test, respectively. The results showed that most key outcome variables—including SMTube, Schirmer, LLTav, TBUT, and OSDI—were not normally distributed (*p* < 0.05), and the assumption of homogeneity of variances was violated. Therefore, nonparametric tests were deemed appropriate and robust for subsequent analyses.

Tear film and meibomian gland parameters were stratified by age, sex, spherical equivalent (SE), and OSDI scores. Group comparisons were performed using the Mann–Whitney *U* test (for two groups) or the Kruskal–Wallis H test (for multiple groups), with post hoc adjustments via the Bonferroni correction. Correlations were evaluated using Spearman’s rank‐order test. A significance level of *α* = 0.05 was applied throughout.

Sample size estimation was based on NIBUT, a parameter with high variability. Based on a standard deviation (σ) of 2.0 s, a margin of error (E) of 0.5 s, *α* = 0.05, and 80% power, G Power estimated a minimum of 62 participants. To accommodate potential attrition (15%–20%), additional outcome parameters, and age stratification, 224 subjects were enrolled.

## 3. Result

### 3.1. Baseline Information

A total of 224 children (448 eyes) were enrolled, 86 males (38.4%) and 138 females (61.6%), aged 7 to 14 years (9.82 ± 1.84 years), with SE ranging from −0.25 to −9.50 D (mean: −2.27 ± 1.63 D). All participants had normal intraocular pressure and a best‐corrected visual acuity of 1.0 or above.

### 3.2. Ocular Surface Parameters and OSDI Score Grading

The ocular surface parameters and OSDI score grading are summarized in Table 1. Data are presented as median (interquartile range): SMTube value: 6.0 (4.6, 8.0) mm, Schirmer I test: 17.0 (13.0, 20.0) mm, NIBUTf: 7.4 (5.2, 9.0) s, NIBUTav: 10.1 (7.1, 13.5) s, TMH: 0.21 (0.18, 0.24) mm, LLTav: 64 (57, 67) nm, Sup‐MGA: 33 (29, 37) %, Inf‐MGA: 21.5 (17, 29) %, BF: 4.1(2.6, 6.8) s, and BQ: 100 (83, 100) %.

**Table 1:** Ocular surface parameters and OSDI score grading of 224 myopic school‐aged children.

Variables	Median (Q1, Q3)	Mean ± SD
Tear film function		
SMTube (mm)	6.0 (4.6.8.0)	6.8 ± 3.1
Schirmer (mm)	17.0 (13.0.20.0)	16.8 ± 4.1
TMH (mm)	0.21 (0.18.0.24)	0.22 ± 0.07
LLTav (nm)	64 (57.67)	62 ± 11
NIBUTf (s)	7.4 (5.2.9.0)	7.5 ± 3.2
NIBUTav (s)	10.1 (7.1.13.5)	10.4 ± 4.1
Meibomian gland atrophy (%)		
Sup‐MGA	33 (29.37)	33.1 ± 6.6
Inf‐MGA	21.5 (17.29)	23.7 ± 9.6
Blink parameters		
BF (20s/blinks)	4.1 (2.6.6.8)	5.0 ± 3.8
BQ (complete/total blinks)	100 (83,100)	91.8 ± 10.3

**OSDI grades**	**N (%)**	

Grade 1 (Asymptomatic)	172 (72.8%)	
Grade 2 (Mild)	29 (12.9%)	
Grade 3 (Moderate)	18 (8.1%)	
Grade 4 (Severe)	5 (2.2%)	

*Note:* Nonparametric data are presented as median (first quartile, third quartile). Values are additionally reported as mean ± standard deviation to enhance clarity.

Abbreviations: BF, blink frequency; BQ, blink quality; Inf‐MGA, inferior meibomian gland atrophy; LLTav, mean lipid layer thickness; NIBUTf, noninvasive 8rst break time; NIBUTav, noninvasive mean break time; OSDI, Ocular Surface Disease Index; SMTube, strip meniscometry tube; Sup‐MGA, superior meibomian gland atrophy; TMH, tear meniscus height.

The OSDI questionnaire score showed that 172 participants (76.8%) were asymptomatic (Grade 0), 29 (12.9%) had Mild Symptoms (Grade 1), 18 (8.1%) had Moderate Symptoms (Grade 2), and 5 (2.2%) had Severe Symptoms (Grade 3).

### 3.3. Differences of Ocular Surface Parameters in Different OSDI Grades

The Kruskal–Wallis test revealed significant differences in age and Schirmer I test values among the OSDI grade groups (both *p* < 0.001). Post hoc analysis showed that participants with Mild Symptoms (Grade 1) and Moderate Symptoms (Grade 2) were significantly older than those who were Asymptomatic (Grade 0). Furthermore, participants with Severe Symptoms (Grade 3) had significantly lower Schirmer I test and SMTube values compared to the asymptomatic group (all *p* < 0.05) (Table 2).

**Table 2:** Differences of ocular surface parameters in different OSDI scores.

OSDI grades	Case	Age (years)	Schirmer (mm)	SMTube (mm)	NIBUTf (s)	TMH (mm)	LLTav (nm)	BF (s)	BQ (100%)	Sup‐MGA (%)	Inf‐MGA (%)
0	172 (76.8%)	9 (8.11)	16 (13.20)c	6 (5.8.75)	7.40 (5.17.9.04)	0.21 (0.18.0.24)	64.5 (57.67)	4.1 (2.6.6.8)	100 (83,100)	33 (29.37)	21 (16.27)
1	29 (12.9%)	10 (9.11.25)a	18 (15.21)d	6 (5.8)	7.28 (5.14.8.84)	0.22 (0.18.0.26)	65 (56.67)	5.2 (3.1.7.0)	100 (80,100)	33 (28.5.37)	25 (17.5.32)
2	18 (8.1%)	11 (9.12)b	18.5 (16.20)e	6 (4.25.8)	7.28 (4.97.8.58)	0.22 (0.18.0.24)	64 (57.2.66)	4.8 (2.4.7.4)	100 (80.5,100)	32 (28.37)	24 (18.31)
3	5 (2.2%)	10 (8.5.10.25)	12 (10.12.5)	4 (3.6)f	9.12 (6.66.9.79)	0.23 (0.20.0.25)	64.5 (61.66)	5.2 (2.5.5.7)	100 (92.3,100)	31 (23.5.37)	23 (18.32)
z	—	16.191	24.725	9.018	4.634	0.665	0.663	2.844	1.085	1.395	7.664
*p*	—	< 0.001^∗∗^	< 0.001^∗∗^	0.029^∗^	0.201	0.881	0.882	0.416	0.781	0.707	0.053

*Note:* Abbreviations are the same as in Table 1. Letters a and b revealed that patients with OSDI Grades 1 (Mild) and 2 (Moderate) were significantly older than those with OSDI Grade 0 (Asymptomatic). Letters c, d, and e indicated that patients with OSDI Grade 3 (Severe) had significantly lower Schirmer values than those in the other groups. Letter f indicated patients with OSDI Grade 3 (Severe) had significantly lower SMTube values than those with OSDI Grade 0 (Asymptomatic). All comparisons were corrected by Bonferroni.

^∗^ *p* < 0.05.

^∗∗^ *p* < 0.001.

### 3.4. Differences in Eye Surface Parameters by Age Groups

A significant difference in TMH was found with *p* = 0.000248 between the two age groups. TMH was significantly higher in children aged 11–14 years than those aged 7–10 (Table 3).

**Table 3:** Differences in eye surface parameters by age groups.

Age (years)	Case	Schirmer (mm)	SMTube (mm)	NIBUTf (s)	NIBUTav (s)	TMH (mm)	LLTav (nm)	BF (s)	BQ (%)	Sup‐MGA (%)	Inf‐MGA (%)
7–10	142 (63.4%)	16 (13.20)	6 (5.8)	7.5 (4.9.9.1)	10.1 (6.7.13.6)	0.21 (0.18.0.24)	64 (56.67)	4.1 (2.6.6.8)	100 (82,100)	33 (29.37)	21 (17.27)
11–14	82 (36.6%)	15 (18.20)	6 (4.8)	7.3 (5.5.9.0)	10.3 (7.7.13.5)	0.22 (0.20.0.26)	65 (59.68)	4.1 (2.8.6.9)	100 (84,100)	33 (29.37)	23 (17.31)
z	—	−1.556	−0.261	−0.23	−1.089	−3.665	−1.803	−0.668	−0.35	−0.313	−1.819
*p*	—	0.12	0.794	0.818	0.276	0.000248^∗^	0.071	0.504	0.726	0.755	0.069

*Note:* Abbreviations are the same as in Table 1. TMH was significantly higher in children aged 11–14 years than those aged 7–10.

^∗^ *p* < 0.05.

### 3.5. Differences in Eye Surface Parameters by Sex

Significant differences in NIBUTf, NIBUTav, and LLTav were found with *p* = 0.018, 0.019, and 0.040 between the sexes. Boys had significantly higher values for these parameters than girls (all *p* < 0.05) (Table 4).

**Table 4:** Differences in eye surface parameters by sex.

Sex	Case	Age (years)	Schirmer (mm)	SMTube (mm)	NIBUTf (s)	NIBUTav (s)	TMH (mm)	LLTav (nm)	BF (s)	BQ (%)	Sup‐MGA (%)	Inf‐MGA (%)
Girl	86 (38.4%)	9 (8.11)	16 (13.20)	5.5 (4.8)	6.8 (4.8,8,8)	9.9 (6.4.13.3)	0.21 (0.18.0.24)	64 (54.66)	4.1 (2.6.6.8)	100 (84,100)	33 (29.38)	23 (18.30)
Boy	138 (61.6%)	10 (9.11)	17 (13.20)	6 (5.8)	7.6 (5.4.9.2)	10.2 (7.6.13.6)	0.22 (0.19.0.25)	65 (58.67)	4.1 (2.8.6.9)	100 (82,100)	32 (29.37)	20 (16.29)
z	—	0.978	0.388	1.871	2.358	2.352	1.702	2.053	0.413	−0.656	−1.944	−1.951
*p*	—	0.328	0.698	0.061	0.018^∗^	0.019^∗^	0.089	0.040^∗^	0.680	0.512	0.052	0.051

*Note:* Abbreviations are the same as in Table 1. Significant differences were observed between the sexes for NIBUTf, NIBUTav, and LLTav. Boys had significantly higher values for these parameters than girls.

^∗^ *p* < 0.05.

### 3.6. Differences in Ocular Surface Parameters by Myopia Degree

Based on the SE, participants were categorized into three myopia groups: mild (−3.00 D ≤ SE ≤ −0.25 D), moderate (−6.00 D ≤ SE  < −3.00 D), and severe (SE < −6.00 D). No significant differences in ocular surface parameters were found between the different myopia groups (Table 5).

**Table 5:** Differences in ocular surface parameters by myopia degree.

Myopia degree	Case	Age (years)	Schirmer (mm)	SMTube (mm)	NIBUTf (s)	NIBUTav (s)	TMH (mm)	LLTav (nm)	BF (s)	BQ (%)	Sup‐MGA (%)	Inf‐MGA (%)
Mild	168 (75%)	9 (8.11)	17 (14.20)	6 (4.6.8)	7.2 (5.1,9,0)	9.9 (7.0.13.6)	0.22 (0.18.0.24)	64 (57.67)	4.1 (2.6.6.9)	100 (82,100)	32 (29.37)	22 (17.29)
Moderate	47 (21%)	9.5 (9.11)	16 (12.19)	6 (4.8)	7.7 (5.7.9.1)	11.0 (8.2.13.6)	0.21 (0.18.0.24)	66 (58.68)	4.2 (3.0.6.8)	100 (86,100)	33 (29.38)	22 (17.32)
Severe	18 (4%)	10 (9.11)	19 (16.21)	6 (5.9)	7.3 (5.2.9.1)	8.3 (6.4.12.8)	0.20 (0.18.0.30)	65 (60.67)	5.2 (3.7.6.8)	100 (82,100)	33 (28.37)	21 (16.29)
z	—	2.888	5.821	1.063	1.952	1.711	0.387	3.070	2.034	1.421	1.710	2.598
*p*	—	0.236	0.054	0.588	0.377	0.425	0.824	0.215	0.362	0.491	0.425	0.337

*Note:* Abbreviations are the same as in Table 1. No significant differences in ocular surface parameters were found between the different myopia groups.

### 3.7. Correlation Between SMTube Values and Other Ocular Surface Parameters

**Figure 1:**
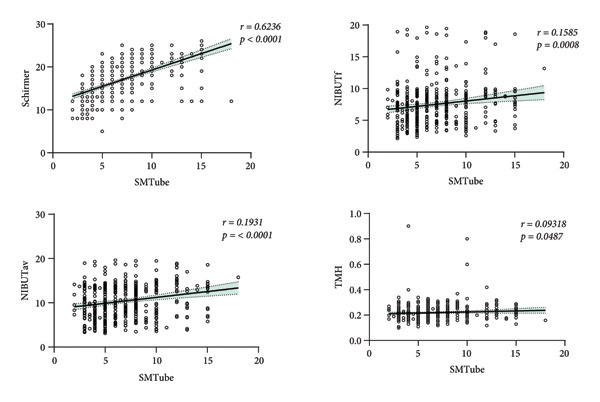
Correlation between SMTube values and other ocular surface parameters.

Correlation analysis revealed positive correlations between SMTube values and Schirmer values, NIBUTf, NIBUTav, and TMH (*r* = 0.6236, 0.1585, 0.1931, and 0.009318, *p* < 0.0001, *p* = 0.0008, and *p* = 0.0487) (Figure 1).

### 3.8. Correlation Between Blinking and Ocular Surface Parameters

No significant correlation was found between BF, BQ, and other ocular surface parameters.

## 4. Discussion

Recently, clinical ophthalmologists have shown increasing attention to the prevalence of dry eye in children. However, the reported prevalence rates vary across studies [13–15]. In this study, the proportion of children with dry eye symptoms (OSDI score ≥ 13) was 23.2%, similar to the 26.6% reported by Uchino et al. [13] but slightly higher than the 17% reported by Tichenor et al. [14]. These variations may be mainly due to the different diagnostic criteria for dry eye across studies. Second, we primarily studied myopic school‐aged children. To date, few studies have focused on dry eyes in myopic children. Finally, factors such as race and age also play a role, with studies consistently reporting a higher prevalence of dry eye among Asian populations [16].

Significant differences in age, SMTube values, and Schirmer scores were observed among the OSDI score groups. The severity of dry eye symptoms in myopic children increased with age, consistent with previous studies [16], and may be related to the increased near‐work and electronic device use, while in adults, dry eye is often associated with evaporative disease, most commonly caused by meibomian gland dysfunction (MGD) [17, 18], and evidence suggests a similar age‐dependent shift in etiology among youth [19]. Our study suggests that dry eye symptoms in myopic children aged 7–14 are primarily associated with insufficient tear volume. This pathophysiology distinguishes them from typical adult presentations.

The SMTube is a relatively novel, rapid, noninvasive, and convenient tool for assessing the tear river volume, with the entire procedure requiring only 5 seconds [20]. Its simplicity and ease of use are of great help to the clinical staff. Previous studies have reported significant differences in SMTube measurements between patients with dry eyes and healthy individuals [21–23]. Several previous studies have demonstrated a high correlation between SMTube and Schirmer I test results [20, 24], BUT [20, 22], and TMH [21, 22], which is consistent with the conclusions of this study. Specifically, we now propose that the strong correlations with established metrics such as Schirmer test and NIBUTf suggest that the SMTube could serve as a rapid, noninvasive, and child‐friendly screening tool for dry eye in pediatric populations. Its ease of use and tolerance among young patients may make it particularly suitable for first‐line assessment in clinical or school settings, helping to identify children at risk who might require more comprehensive evaluation. However, we also note that further validation in larger and more diverse cohorts is needed to establish definitive diagnostic cut‐offs and evaluate its cost‐effectiveness compared to existing methods.

In this study, the median NIBUTf was 7.4 s, which is lower than the previous studies [25–27], such as Jones et al. [26], who measured a healthy NIBUTf of (21.76 ± 4.06) seconds, but similar to Ma et al. [15]. This discrepancy may be attributed to two key factors. First, this study included myopic children, but Jones et al. [26] studied healthy children; myopia may also be a factor affecting dry eye [4, 5]. Second, ethnic reasons may be due to a meta‐analysis conducted by Chidiegboka et al. [27], which showed that Asian children had relatively low BUT values.

Although myopia may influence dry eye [4, 5], this study found no relationship between the degree of myopia and ocular surface parameters, consistent with the results of previous study by Zhang et al. [28]. However, Ilhan et al. [29] reported a higher incidence of dry eye in adults with high myopia (SE < −6.0 D). The absence of a significant correlation may be attributed to several factors. First, the study population consisted primarily of school‐aged children (7–14 years old) with early‐stage myopia, the majority of whom had low to moderate myopia, while high myopia represented only a small proportion (4%). It is possible that structural or functional changes in the ocular surface related to myopia are not yet pronounced at this age and severity level. Second, environmental and behavioral factors—such as screen time, reading habits, and blink patterns—may exert a more direct influence on tear film stability than refractive error itself in this young cohort. These shared lifestyle factors across myopia severities could attenuate observable differences in ocular surface parameters. Finally, the limited sample size in the high myopia subgroup may have reduced the statistical power to detect a modest association, should one exist.

Sex hormones, particularly androgens, are critical regulators of both the aqueous and lipid layers of the tear film, modulating secretion from the lacrimal and meibomian glands and influencing ocular surface inflammation [30]. We observed higher tear film stability in boys aged 7–14 compared to girls, coinciding with pubertal onset and dynamic hormonal changes. Elevated androgen levels in boys may promote ocular surface health, whereas in girls, a relative estrogen‐androgen imbalance may suppress meibomian lipid secretion and increase tear evaporation [31]. Thus, rather than directly causing dry eye, sex hormones likely promote a susceptible ocular environment that enhances vulnerability to external stressors such as reduced BF.

TMH, an indicator of tear volume, was measured with a median of 0.21 mm in this study, a value consistent with some reports [32], but slightly lower than 0.3 mm in Akil’s study [33]. Older children (11–14 years) exhibited significantly higher TMH than younger children (7–10 years), consistent with the reported findings that tear secretion peaks between 10 and 20 years of age before declining significantly [34]. The increase in TMH during adolescence corresponds to the peak period of myopia onset and progression. We hypothesize that elevated TMH may enhance ocular surface hydration during axial elongation, representing a potential compensatory response or a concurrent process related to pubertal changes. Thus, TMH could serve as an auxiliary indicator of myopia risk, though longitudinal studies are necessary to establish a causal relationship with axial growth. Furthermore, the elevated tear volume characteristic of this high‐secretion phase may mitigate dry eye symptoms under conditions of high visual demand, thereby enhancing ocular surface resilience. Adolescents reporting significant dry eye symptoms should therefore be evaluated for underlying conditions, including meibomian gland dysfunction, autoimmune disorders, or excessive screen use.

A large majority of children in this study (81.7%) exhibited an average lipid layer thickness (LLTav) below the 75 nm cut‐off for MGD [35], a proportion similar to that reported by Chidi‐Egboka et al. [36]. However, this was not associated with dry eye symptoms or reduced meibomian gland expression capacity, contrasting with the significant associations observed in adults [35, 37]. We also observed a relatively high degree of MGA in both the upper and lower eyelids. Collectively, these alterations in gland morphology and function are increasingly recognized as a normative finding in pediatric populations, with previous studies noting that such changes can manifest in children as young as 6 years old [36, 38].

Previous studies have found that the incomplete blink rate is significantly correlated with the meibomian gland area ratio and dry eye symptoms [39]. In this study, BF and BQ showed no significant correlation with other ocular surface parameters or dry eye discomfort. This discrepancy may be because Jeon’s study population is adults, with an average age of 56.8 ± 14.5 years [39], while our study focused on children with a mean age of 9.82 ± 1.84 years. Age and systemic and psychological factors may contribute to these differing conclusions.

In summary, dry eye symptoms are prevalent in school‐aged children with myopia. Age and sex are key determinants of tear film quality, with tear deficiency being the primary cause of discomfort. Myopia severity showed no significant association with tear film parameters. The SMTube shows promise as a rapid, noninvasive, child‐friendly screening tool. These findings highlight the need to integrate ocular surface health assessments into routine pediatric myopia management.

This study has several limitations. First, the study sample was primarily recruited from a single pediatric optometry clinic. While this provided a well‐characterized cohort, it may limit the generalizability of our findings to the broader pediatric population, and future studies should aim to include children from more diverse settings, such as schools and general pediatric practices, to confirm and extend our results. Second, the distribution of gender and age groups was unequal as our department is a pediatric optometry clinic, and a slightly higher proportion of girls (7–10) presented for initial visits, which may affect the analysis results. Finally, there are currently no definitive diagnostic criteria for dry eye in children. However, we believe the comprehensive ocular surface parameters presented in this study provide a valuable foundation for advancing dry eye diagnosis in children.

## Conflicts of Interest

The authors declare no conflicts of interest.

## Author Contributions

Study concept and design: Jiwen Yang and Limin Bu. Patients’ recruitment and clinical data collection: Haiyun Wang, Yanwen Zheng, and Guang Wang. Analysis and interpretation of data: Haiyun Wang and Yanwen Zheng. Writing the manuscript: Haiyun Wang. Jiwen Yang was responsible for the final approval of the version of this work submitted for publication and agreed to be accountable for all aspects of the work.

## Funding

This study was funded by the Clinic Research Foundation of Aier Eye Hospital Group (Grant No. AGK2308D01).

## Data Availability

More data, if necessary, are available from the corresponding author on reasonable request.
